# Cyst formation in proximal renal tubules caused by dysfunction of the microtubule minus-end regulator CAMSAP3

**DOI:** 10.1038/s41598-021-85416-x

**Published:** 2021-03-12

**Authors:** Yuto Mitsuhata, Takaya Abe, Kazuyo Misaki, Yuna Nakajima, Keita Kiriya, Miwa Kawasaki, Hiroshi Kiyonari, Masatoshi Takeichi, Mika Toya, Masamitsu Sato

**Affiliations:** 1 Laboratory of Cytoskeletal Logistics, Department of Life Science and Medical Bioscience, Graduate School of Advanced Science and Engineering, Waseda University, 2-2 Wakamatsucho, Shinjuku-ku, Tokyo, 162-8480 Japan; 2 Laboratory for Animal Resources and Genetic Engineering, RIKEN Center for Biosystems Dynamics Research, Kobe, 650-0047 Japan; 3 Ultrastructural Research Team, RIKEN Center for Life Science Technologies, Kobe, 650-0047 Japan; 4 Laboratory for Cell Adhesion and Tissue Patterning, RIKEN Center for Biosystems Dynamics Research, Kobe, 650-0047 Japan; 5 Major in Bioscience, Global Center for Science and Engineering, Faculty of Science and Engineering, Waseda University, 3-4-1 Okubo, Shinjyuku-ku, Tokyo, 169-8555 Japan; 6 Institute for Advanced Research of Biosystem Dynamics, Waseda Research Institute for Science and Engineering, Graduate School of Advanced Science and Engineering, Waseda University, 3-4-1 Okubo, Shinjuku-ku, Tokyo, 169-8555 Japan; 7 Institute for Medical-Oriented Structural Biology, Waseda University, 2-2 Wakamatsucho, Shinjuku-ku, Tokyo, 162-8480 Japan

**Keywords:** Microtubules, Polycystic kidney disease

## Abstract

Epithelial cells organize an ordered array of non-centrosomal microtubules, the minus ends of which are regulated by CAMSAP3. The role of these microtubules in epithelial functions, however, is poorly understood. Here, we show that the kidneys of mice in which *Camsap3* is mutated develop cysts at the proximal convoluted tubules (PCTs). PCTs were severely dilated in the mutant kidneys, and they also exhibited enhanced cell proliferation. In these PCTs, epithelial cells became flattened along with perturbation of microtubule arrays as well as of certain subcellular structures such as interdigitating basal processes. Furthermore, YAP and PIEZO1, which are known as mechanosensitive regulators for cell shaping and proliferation, were activated in these mutant PCT cells. These observations suggest that CAMSAP3-mediated microtubule networks are important for maintaining the proper mechanical properties of PCT cells, and its loss triggers cell deformation and proliferation via activation of mechanosensors, resulting in the dilation of PCTs.

## Introduction

Epithelial cells are major components of various organs. They show apical-to-basal polarity that is linked with their functions, such as absorption and secretion. In differentiated epithelial cells, the majority of microtubules are non-centrosomal and are aligned along the apical-to-basal axis^1–3^. CAMSAP3 is a member of the calmodulin-regulated spectrin-associated protein (CAMSAP)/Nezha/Patronin protein family and is important for epithelial microtubule organization^4–8^. CAMSAP3 binds and stabilizes the minus ends of microtubules^4,6,9^. In cells of the small intestine in mice, CAMSAP3 is concentrated at apical cortical regions, anchoring the minus ends of non-centrosomal microtubules to these sites^10,11^, resulting in formation of microtubule arrays oriented toward the apico-basal direction with the plus ends pointed basally. However, this characteristic microtubule organization is lost in mice bearing a mutant gene encoding a truncated *Camsap3* (*Camsap3*^*dc/dc*^ mice), in which the C-terminal microtubule-binding domain is deleted. Intestinal epithelial cells in these mice have various cytological defects, including reduced cell height and mispositioned organelles such as the nucleus and Golgi^10^. Despite such large perturbations in intracellular architecture, the epithelial cells maintain normal-looking two-dimensional sheets^10,12^, and the mutated intestines had no obvious physiological defects.

The kidney is a typical epithelial organ. The nephron, the fundamental structural and functional unit of the kidney, is composed of a renal corpuscle and tubules. The renal tubules are subdivided into proximal convoluted tubules (PCTs), the loop of Henle, distal convoluted tubules, and collecting duct. These nephron substructures consist of a single layer of epithelium, which functions for filtration, reabsorption, and secretion.

Renal cyst is a common pathological lesion of the kidneys. The cystic lesion is variable; either focal or multiple cysts form in one side or bilaterally in the kidneys, showing a different degree of symptoms^13–15^. Several congenital cystic renal diseases, including polycystic kidney diseases (PKD), glomerulocystic kidney disease (GCKD) and medullary cystic kidney disease (MCKD), are known to cause multiple renal cysts in bilateral kidneys. In PKD, cysts are distributed uniformly over the entire organ including PCTs, whereas GCKD and MCKD develop cysts in Bowman’s capsules and collecting ducts (mainly at the medulla region), respectively^16–19^.

As for PKD, two major causes are known: 1) mutations in the gene *Pkd1* or *Pkd2*, and 2) mutations in genes required for primary cilium biogenesis. *Pkd1* and *Pkd2* encode polycystin1 and polycystin2, respectively, which are non-selective cation channels^20^ that localize at several cellular compartments including the primary cilia^21,22^. In mice carrying a mutant form of PKD1 or PKD2, polycystin proteins do not localize to cilia^23^. Genes required for biogenesis of primary cilia include *Ift88* and *Kif3a*, which are involved in intraflagellar transport^24–26^. Mutations in these genes also result in renal cystic diseases^24–27^. Although the relationship between these two causal pathways is complicated^23^, one pathway that has been demonstrated to induce PKD involves the Hippo signaling effector Yap^28^. In *Pkd1* mutant mice, the transcription co-activator YAP/TAZ and its effector c-Myc are activated and promote cystogenesis^28^. Nevertheless, the mechanisms underlying the development of polycystic kidneys remain largely unknown.

In the present study, we investigated the roles of CAMSAP3 in kidney structure and function. Our observations show that apically localized CAMSAP3 contributes to proper orientation of non-centrosomal microtubules as well as establishment of the intracellular architecture of epithelial cells in PCTs, as observed in the small intestine. Unlike the intestinal epithelia, however, CAMSAP3 dysfunction caused organ-level abnormalities in bilateral kidneys—that is, cyst formation in PCTs. Loss of CAMSAP3 function resulted in activation of mechanosensitive regulators that normally help preserve tissue integrity. Our study suggests that CAMSAP3-mediated non-centrosomal microtubule organization is important for maintaining the mechanosensitive properties of PCTs, and loss of this system causes regional cyst formation in the kidney.

## Results

### Cyst formation in kidneys of *Camsap3* mutant mice

**Figure 1:**
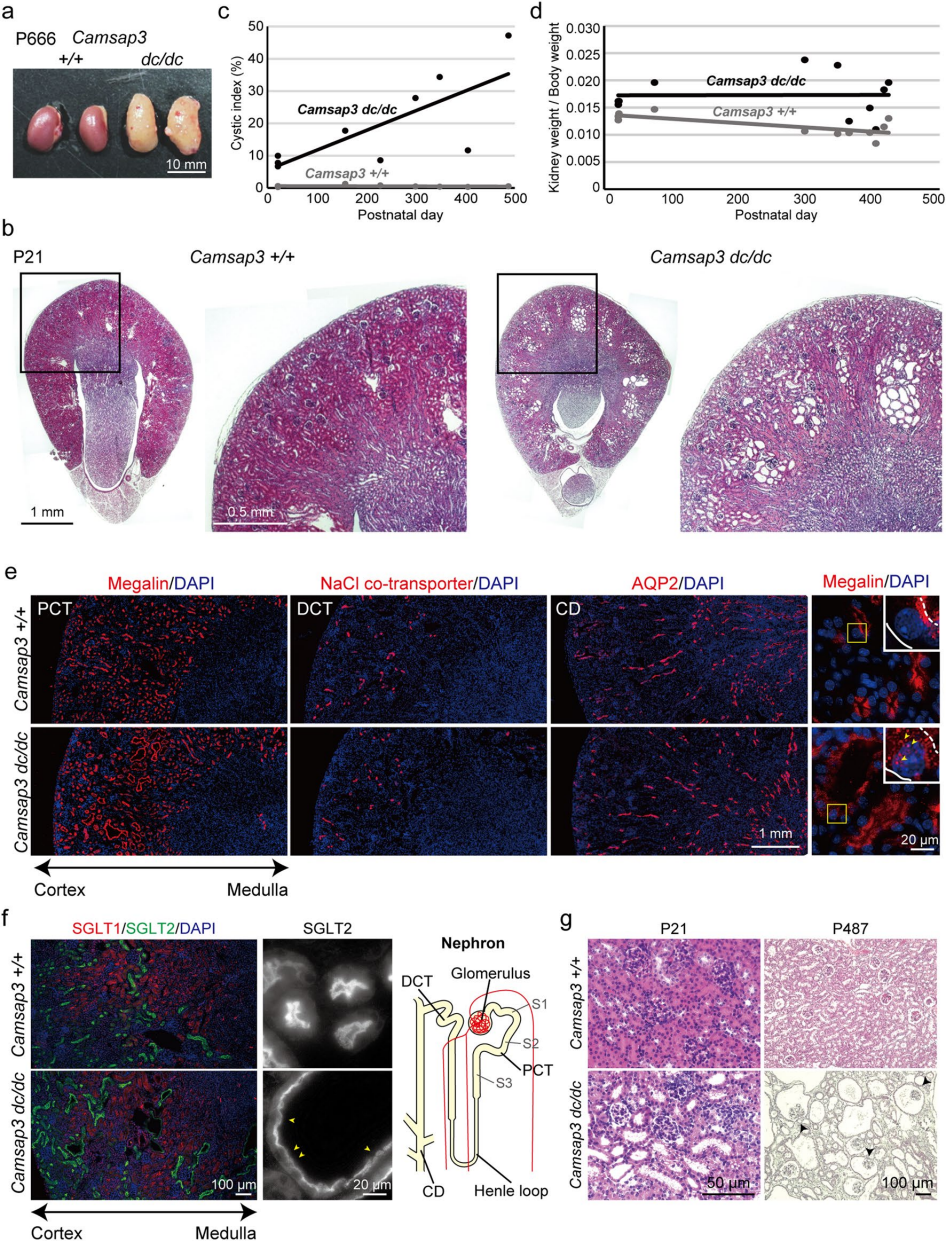
Cyst formation in proximal convoluted tubules of *Camsap3*^*dc/dc*^ kidneys. (**a**) Morphology of kidneys of WT (+*/*+) and *Camsap3* mutant (*Camsap3*^*dc/dc*^) mice at P666. (**b**) H&E staining of WT and *Camsap3*^*dc/dc*^ kidneys at P21. Multiple cysts formed in the cortex of *Camsap3*^*dc/dc*^ kidneys. (**c**) Change of cystic index of WT and *Camsap3*^*dc/dc*^ mice over time. 9 mice were examined for WT or *Camsap3*^*dc/dc*^. See the MATERIALS AND METHODS for the definition of cystic index. (**d**) Ratio of kidney to body weight. 11 mice were examined for WT or *Camsap3*^*dc/dc*^. (**e**) Immunostaining for markers of proximal convoluted tubules (PCT) (Megalin), distal convoluted tubules (DCT), NaCl co-transporter, and collecting ducts (CD) (aquaporin-2; AQP2) in WT and *Camsap3*^*dc/dc*^ kidneys at P21. Images of the magnified view of Megalin show delocalized Megalin in cystic cells in *Camsap3*^*dc/dc*^ kidneys, as indicated by the arrowhead. Broken and continuous lines indicate the apical and basal margin of the cell, respectively. (**f**) Immunostaining for markers of the S1/S2 segments (SGLT2, green) and S3 segment (SGLT1, red) in WT and *Camsap3*^*dc/dc*^ kidneys at P21. Images of the magnified view of SGLT2 show decreased SGLT2 signal intensity in cystic cells in *Camsap3*^*dc/dc*^ kidneys, as indicated by arrowheads. (**g**) Morphology of the glomerulus at P21 and P487. Arrowheads indicate the dilated glomerulus. See also Figures S1 and S2.

Phenotypic analysis of *Camsap3*^*dc/dc*^ mutant mice revealed that kidneys in aged (~ 2 years old) *Camsap3*^*dc/dc*^ mice were hypertrophic and discolored (Fig. 1a). To further analyze abnormalities in *Camsap3*^*dc/dc*^ kidneys, we examined younger mice. At postnatal day 21 (P21), *Camsap3*^*dc/dc*^ kidneys had a normal appearance. However, sectioning of the kidney revealed cystic structures at the cortical regions (Fig. 1b and Supplementary Fig. S1a), as confirmed by calculating the cystic index, which is the ratio of the cyst-containing areas to the total kidney area (Fig. 1c). We examined mutant embryos to determine when cysts begin to form during development, which revealed a sign of increased cyst formation at approximately embryonic day 17.5 (E17.5) (Supplementary Fig. S1a, arrows). At P0, cortical cysts became readily discernable (Supplementary Fig. S1a, arrows), and their size and number had increased by P21 (Fig. 1b, Supplementary Fig. S1a, arrows). As mice grew older, cystic phenotypes became more evident, although the degree of cyst formation varied among the individuals (Fig. 1c and Supplementary Fig. S1b). In addition, the kidney-to-body weight ratio was higher for *Camsap3*^*dc/dc*^ mice than wild-type (WT) mice over their lifetime (Fig. 1d and Supplementary Fig. S1c, d). Thus, *Camsap3*^*dc/dc*^ kidneys exhibited cyst formation, which was enhanced over time, resulting in hypertrophy in aged kidneys.

*Camsap3*^*dc/dc*^ mice express a C-terminal domain–truncated CAMSAP3 (CAMSAP3-dc)^10^, which could be involved in the observed cystic phenotype of the mouse kidneys. To investigate this possibility, we generated a *Camsap3*^*–/–*^ (*Camsap3-null*) mouse (Supplementary Fig. S2a,b) (see also Methods in detail). These mice also had kidneys with multiple cysts, and their phenotype was indistinguishable from that observed in *Camsap3*^*dc/dc*^ mice; notably, heterozygous (*Camsap3*^+*/–*^) mice showed no signs of polycystic kidneys (Supplementary Fig. S2c).

### Cyst formation in PCTs of ***Camsap3***^***dc/dc***^ kidneys

To determine which structures became cystic in the mutant kidneys, we carried out immunostaining of three nephron segments for three proteins, namely Megalin (localizes to PCTs), a Na^+^/Cl^–^ cotransporter (distal convoluted tubules), and aquaporin-2 (collecting ducts). The results showed that dilation occurred exclusively at Megalin-positive tubules at P21 (Fig. 1e and Supplementary Fig. S1e), indicating that cystogenesis was restricted to PCTs. PCTs are subdivided into three segments, namely S1, S2 and S3, and we distinguished between them by immunostaining for the glucose transporters SGLT1 and SGLT2, which localize at S3 and S1/S2 segments, respectively^29,30^. The dilated tubules were marked solely by SGLT2 (Fig. 1f), indicating that cystogenesis occurs at the S1/S2 segments. In older mutant mice, however, cyst formation also extended to the Bowman’s capsule (Fig. 1g).

### Disorganized cellular architecture in cystic PCTs

**Figure 2:**
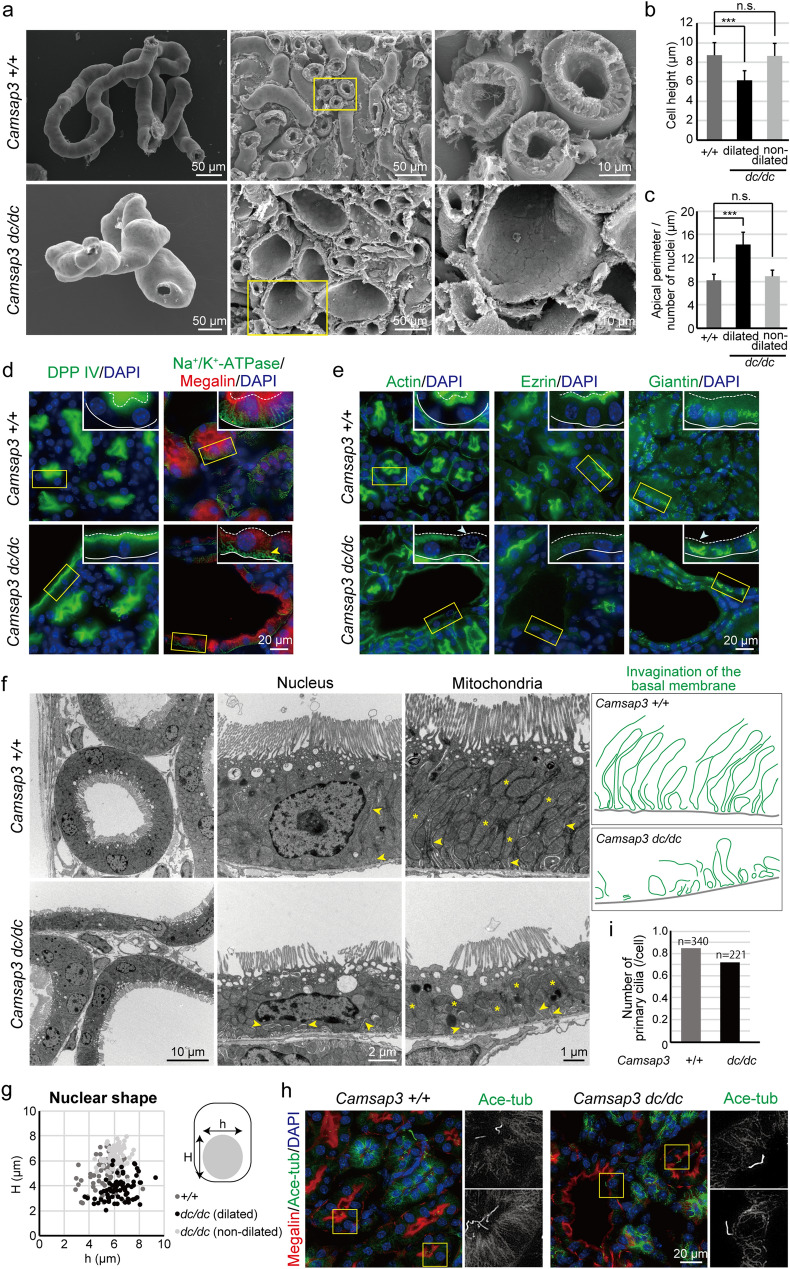
Disorganized cellular architecture in cystic PCT cells. (**a**) Scanning electron microscopy images of WT and *Camsap3*^*dc/dc*^ kidneys at P21. The renal tubule in *Camsap3*^*dc/dc*^ kidneys is partially swollen. (**b**) Quantification of cell height in the cortex [+ */* + , n = 121 cells; *dc/dc* (dilated), n = 128 cells; *dc/dc* (non-dilated), n = 66 cells]. Error bars indicate S.D. ***p < 0.001, t-test. See MATERIALS AND METHODS for the definition of dilated and non-dilated tubules. (**c**) Quantification of cell width in the cortex [+ */* + , n = 26 tubules; *dc/dc* (dilated), n = 8 tubules; *dc/dc* (non-dilated), n = 14 tubules]. Error bars indicate S.D. ***p < 0.001, t-test. (**d**) Cell polarity markers were visualized: DPP IV (apical marker) and Na^+^/K^+^-ATPase (basal marker). The localization of cell polarity markers in *Camsap3*^*dc/dc*^ kidney is overall normal. The arrowhead indicates basal plasma membranes, whose invagination is not normal. (**e**) Apical structures (actin and ezrin) and the Golgi were visualized. Arrowheads indicate disrupted structures such as the lack of actin signal and misplacement of the Golgi. Broken and continuous lines indicate the apical and basal margin of the cell, respectively, in D and E. (**f**) Transmission electron microscopy images of WT and *Camsap3*^*dc/dc*^ kidneys at P21. Subcellular defects such as a flattened nucleus and misaligned mitochondria are observed in *Camsap3*^*dc/dc*^ kidneys. Arrowheads indicate invaginated basal membranes. Note that their extensive invagination is suppressed in *Camsap3*^*dc/dc*^. Asterisks denote mitochondria. Drawings are the traced invagination in electron microscopy images. (**g**) Quantification of the shape of the nucleus [+ */* + , n = 72 cells; *dc/dc* (dilated), n = 72 cells; *dc/dc* (non-dilated), n = 71 cells]. (**h**) Immunostaining for acetylated tubulin, a marker for the primary cilium, in WT and *Camsap3*^*dc/dc*^ kidneys at P21. The primary cilium was present in both WT and *Camsap3*^*dc/dc*^ kidneys. (**i**) Number of primary cilia at PCTs. Number of primary cilia was calculated using the apical circumference length of cross sections of PCT, along with numbers of primary cilia and nuclei. [+/+, N = 340 cells, 63 cross sections of PCT, No. of primary cilia = 268; *dc/dc*, N = 221 cells, 29 cross sections of PCT, No. of primary cilia = 133].

To explore cellular-level defects in cystic PCTs, we first examined them by scanning electron microscopy, finding that a population of renal tubules markedly swelled in *Camsap3*^*dc/dc*^ kidneys (Fig. 2a), although the extent of swelling varied from position to position of the tubule. Cross sections revealed that, at swelled portions of a tubule, its lumen was expanded along with thinning of the epithelial layer composing the tubules (Fig. 2a). Quantification using hematoxylin and eosin–stained samples confirmed that cell height was reduced in the dilated tubules (Fig. 2b), whereas the apical perimeter of each cell was increased (Fig. 2c), indicating that the mutant cells were flatter than those in WT kidneys.

We next examined whether cell architecture and/or subcellular structures of PCTs were affected by *Camsap3* mutation. Immunostaining for DPP IV, an apical membrane marker, and for Na^+^/K^+^-ATPase, a basolateral membrane marker, indicated that the overall cell polarity was normal in every renal tubule cell of *Camsap3*^*dc/dc*^ mice (Fig. 2d). In contrast, although Megalin accumulated at the apical regions in WT cells, it diffused into the cytoplasm in some of the cystic *Camsap3*^*dc/dc*^ cells (Fig. 1e magnified view, and Fig. 2d). Fluorescence staining for F-actin, which accumulates at high density in apical microvilli, suggested that the microvilli were occasionally disarranged in the flattened cells of mutant kidneys (Fig. 2e arrowhead), as confirmed by transmission electron microscopy (Fig. 2f). Ezrin, a part of the ERM-complex which associates with the apical plasma membrane^31^, was decreased or lost in cystic cells of *Camsap3*^*dc/dc*^ kidneys (Fig. 2e), suggesting that cytoarchitecture was abnormal in mutant cells. In addition, whereas the Giantin-positive Golgi apparatus is generally distributed as thin strands juxtaposed to the nucleus in WT cells, the Golgi occasionally became condensed in *Camsap3*^*dc/dc*^ cells (Fig. 2e).

Transmission electron microscopy revealed additional subcellular defects in dilated PCTs of *Camsap3*^*dc/dc*^ kidneys. In WT PCT cells, mitochondria were elongated, showing an ordered orientation along the apico-basal axis of the cell, whereas in cystic PCT cells, mitochondria tended to be round —no longer displaying any ordered orientation (Fig. 2f). In these cells, the basal striations were also disorganized, losing their parallel and deep invaginations that are common in WT cells (Fig. 2f arrowheads and traced drawing), as also shown by immunostaining for Na^+^/K^+^-ATPase (Fig. 2d arrowheads). Moreover, nuclei were flattened in the cystic cells, compared with their round shape in WT cells (Fig. 2f,g). Primary cilia were detectable at the luminal side of PCT cells in both WT and mutant kidneys (Fig. 2h,i), unlike the case for mutant mice that are defective for ciliogenesis, which causes polycystic kidneys^24–27^, suggesting that impairment of ciliogenesis is not the likely reason for the observed polycystic phenotypes in *Camsap3*^*dc/dc*^ mice. Collectively, these observations indicated that dysfunction of CAMSAP3 perturbs the subcellular architecture of PCT cystic cells.

### ***Camsap3***^***dc/dc***^ mice show some physiological defects

We next examined whether *Camsap3*^*dc/dc*^ kidneys had any physiological defects. The aforementioned immunocytological studies showed that Megalin, which functions as a multiligand endocytic receptor^32,33^, tended to diffuse into the cytoplasm of *Camsap3*^*dc/dc*^ PCT cells (Fig. 1e arrowhead, and Fig. 2d), in contrast to its clear apical localization in WT PCT cells. Furthermore, although the glucose transporter SGLT2, which is responsible for 90% of glucose reabsorption^29^, localized primarily at apical regions of *Camsap3*^*dc/dc*^ cells (as also seen in WT cells), its signal intensity decreased in certain regions of cystic tubules (Fig. 1f arrowheads), suggesting that the physiology of the mutant tubules could have been negatively affected. Therefore, we conducted urine and blood tests, using P396- to P424-old mice. Urine tests revealed a tendency of excessive outflow of K^+^, Mg^2+^, and urea nitrogen in *Camsap3*^*dc/dc*^ mice (Supplementary Fig. S3a). Blood tests more convincingly demonstrated that the *Camsap3*^*dc/dc*^ mice had significantly lower levels of K^+^ and Mg^2+^ compared with WT or *Camsap3*^+*/dc*^ mice, although no change was detected in the levels of urea nitrogen and glucose (Supplementary Fig. S3b). Urine and blood creatinine levels did not differ between WT and mutant mice (Supplementary Fig. S3c). Thus, we observed only partial physiological defects in *Camsap3*^*dc/dc*^ mice.

### Microtubule organization in renal tubule epithelial cells

**Figure 3:**
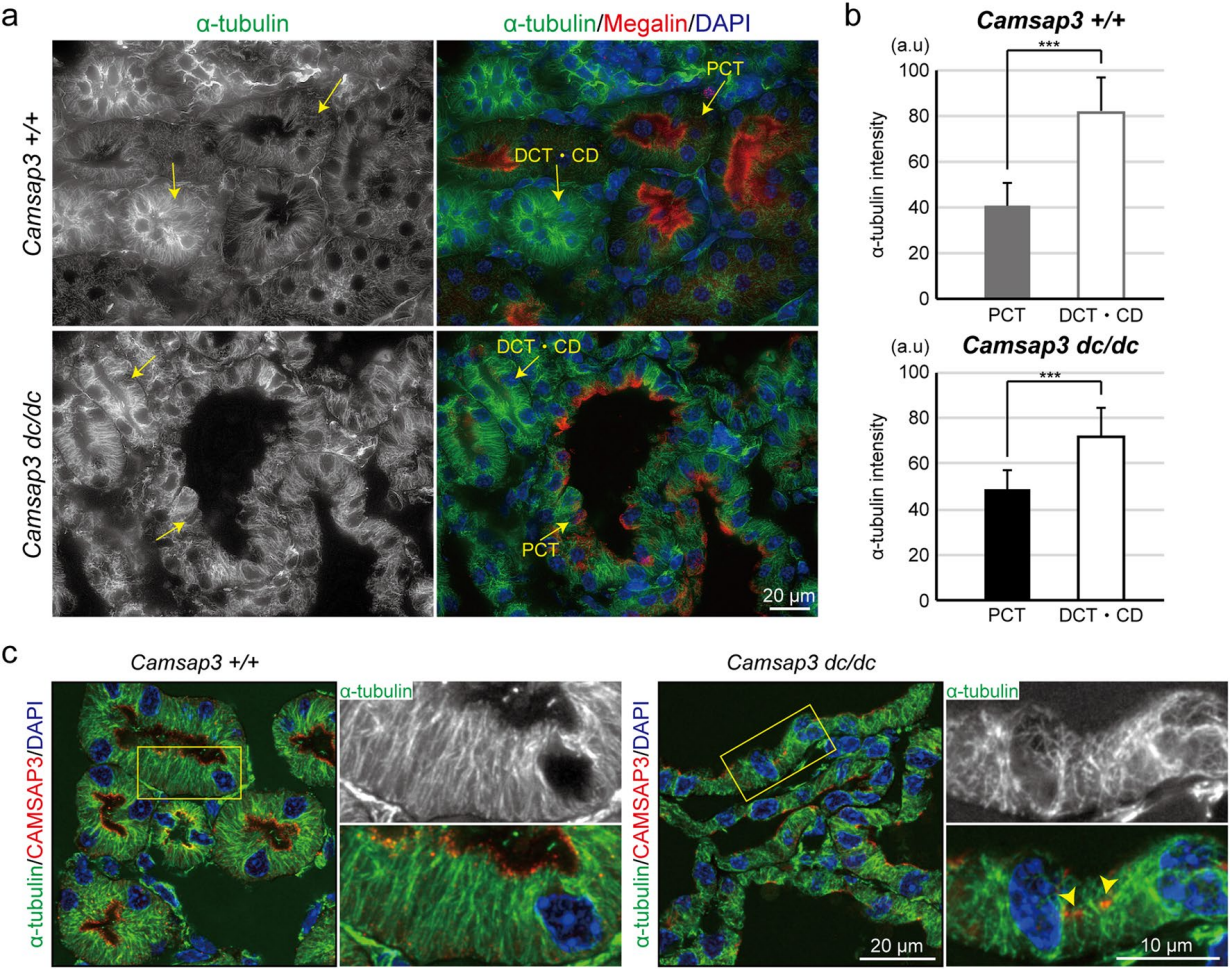
Microtubules in renal tubule epithelial cells. (**a**) Immunostaining for α-tubulin, Megalin, and nuclei in WT and *Camsap3*^*dc/dc*^ kidneys at P21. (**b**) Quantification of α-tubulin intensity in PCT and DCT/CD of WT kidneys (PCT, n = 28 tubules; DCT/CD, n = 18 tubules) and *Camsap3*^*dc/dc*^ kidneys (PCT, n = 13 tubules; DCT/CD, n = 9 tubules). Error bars indicate S.D. ***p < 0.001, t-test. (**c**) Immunostaining for α-tubulin, CAMSAP3, and DAPI in WT and *Camsap3*^*dc/dc*^ kidneys at P21. Arrowheads indicate CAMSAP3-dc proteins anchoring to unidentified structures. The apical localization of CAMSAP3 was absent in *Camsap3*^*dc/dc*^ cells. See also Figure S2D.

As *Camsap3* mutation leads to disorientation of microtubules in epithelial cells of the small intestine^10,12^, we examined whether this also occurs in kidney epithelial cells. We first immunostained α-tubulin together with Megalin. Notably, both in WT and *Camsap3*^*dc/dc*^ kidneys, Megalin-negative tubules, that is, either distal convoluted tubules or collecting ducts, showed stronger α-tubulin immunostaining intensity than observed for α-tubulin in Megalin-positive PCTs (Fig. 3a, b). As found in the small intestine^10^, CAMSAP3 localized apically in all epithelial cells of WT kidneys, overlapping the apical end of microtubules that are aligned along the apico-basal axis (Fig. 3c left). *Camsap3*^*dc/dc*^ kidney epithelial cells also were somewhat immunopositive for CAMSAP3 but at irregular positions (Fig. 3c right, arrowheads), suggesting that mutated CAMSAP3 is retained in the cells, anchored to some unidentified structures. In cystic cells of the mutant kidneys, the array of microtubules was tangled and disorganized (Fig. 3c right), as observed in intestinal cells of *Camsap3*^*dc/dc*^ mice^10^. In non-cystic cells, however, microtubule organization was not affected much even in the absence of functional CAMSAP3, which may explain why these cells showed no cytological abnormalities. In addition, *Camsap3-*null kidney epithelial cells also showed similar microtubule defects but only in cystic cells (Supplementary Fig. S2d). Collectively, these results indicated that CAMSAP3 plays a role in maintaining the apico-basal organization of microtubules only for a subpopulation of kidney epithelial cells.

### Enhanced cell proliferation in ***Camsap3***^***dc/dc***^ PCTs

**Figure 4:**
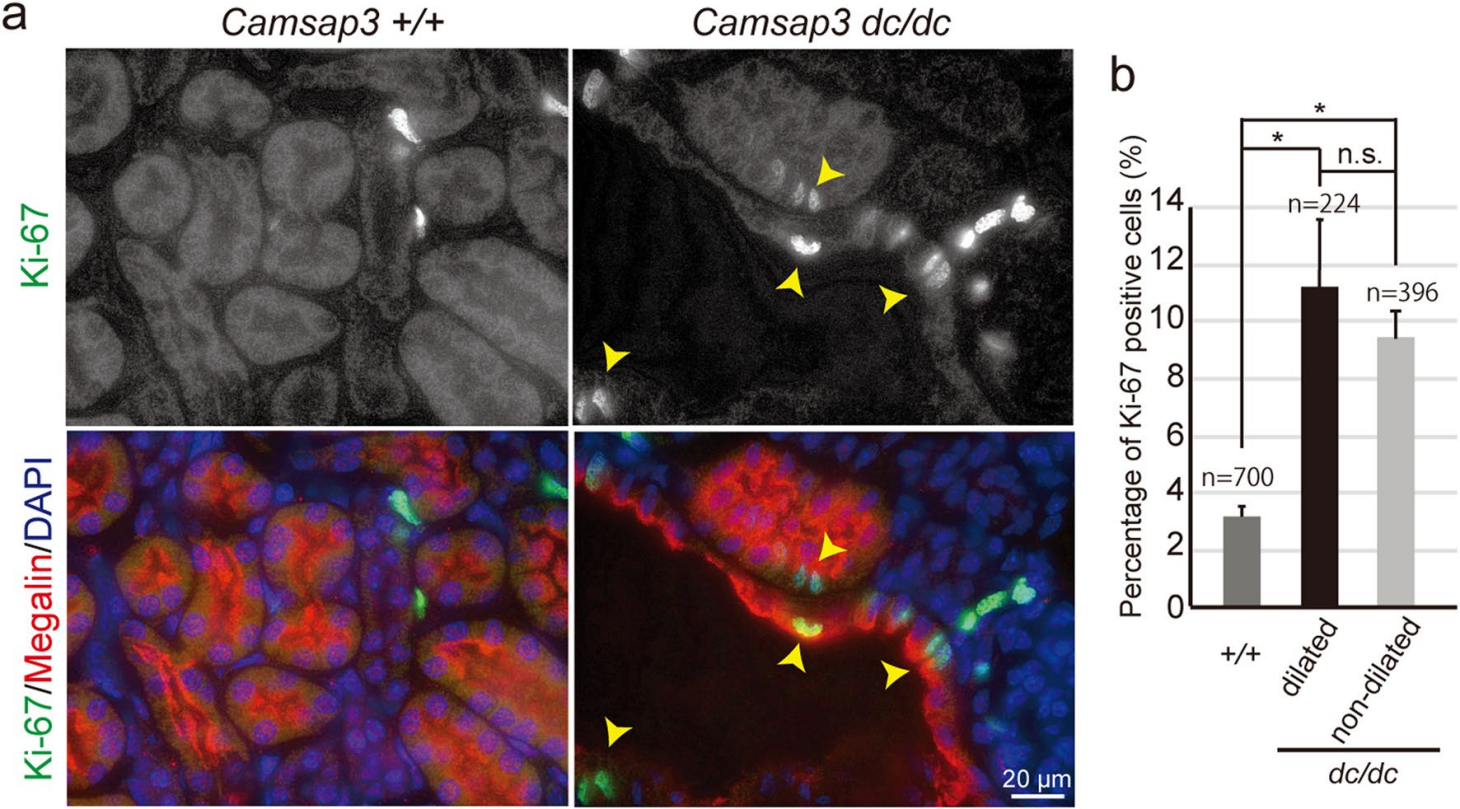
Cell proliferation in *Camsap3*^*dc/dc*^ PCTs. (**a**) Immunostaining for Ki-67, Megalin, and DAPI in WT and *Camsap3*^*dc/dc*^ kidneys at P22. Arrowheads indicate Ki-67–positive cells in cystic tubules. (**b**) Percentage of Ki-67–positive cells within PCT epithelial cells [+*/*+, n = 700 cells; *dc/dc* (dilated), n = 224 cells; *dc/dc* (non-dilated), n = 396 cells]. Error bars indicate S.D. *p < 0.05, t-test.

Cyst formation in kidneys is closely related to enhanced renal cell proliferation^34^. To test whether cell proliferation was altered in *Camsap3*^*dc/dc*^ kidneys, we compared the proliferation of PCT cells between WT and mutant kidneys by co-immunostaining for Megalin and Ki-67, a marker for cell proliferation. Ki-67–positive cells were rarely observed in PCTs of WT kidneys, whereas they were readily apparent in PCT cells of *Camsap3*^*dc/dc*^ kidneys. However, Ki-67 was detected in both non-dilated and dilated tubules (Fig. 4a,b), showing no correlation between cell proliferation and cyst formation. Thus, CAMSAP3 dysfunction did promote cell proliferation, but this process was not always associated with cyst formation.

### Changes in localization of YAP and non-muscle myosin linked with cell deformation

**Figure 5:**
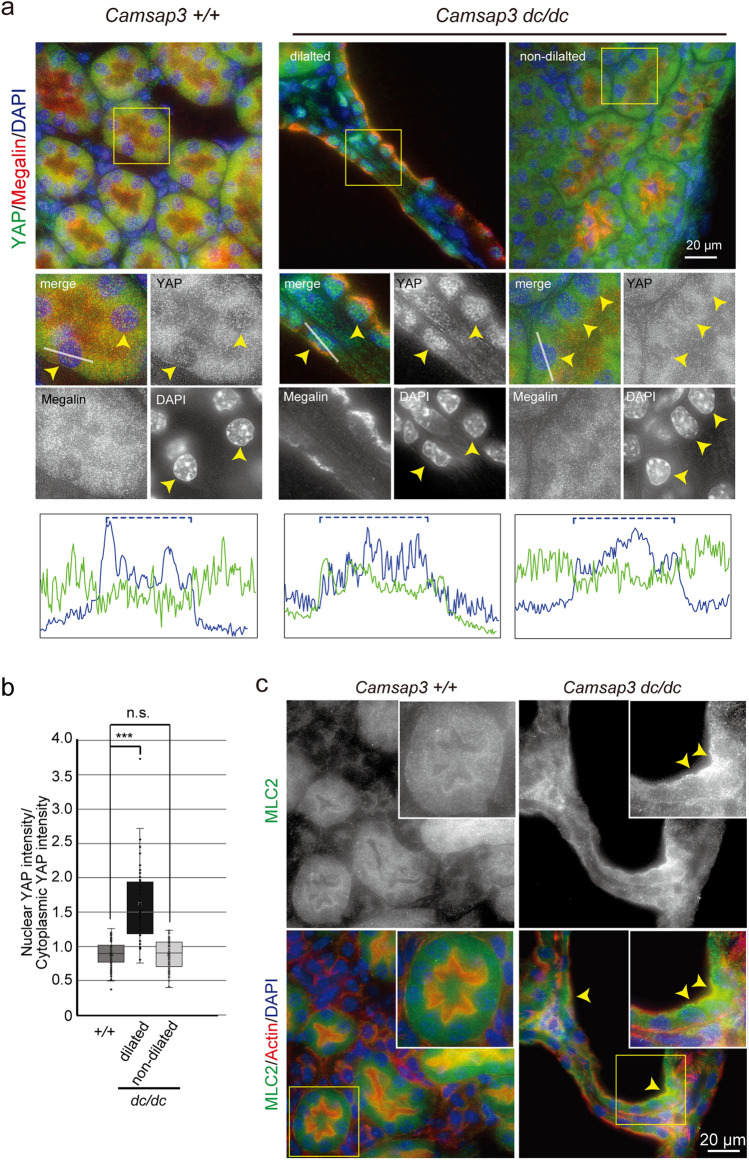
Localization of YAP and non-muscle myosin associated with cell deformation. (**a**) Immunostaining for YAP, Megalin, and DAPI in WT and *Camsap3*^*dc/dc*^ kidneys at P22. Fluorescence signals of DAPI and YAP were scanned along the line drawn in the enlarged images, and the spatial changes in fluorescence intensity are shown in blue for DAPI and green for YAP. Dashed lines indicate the approximate area of the nucleus. Arrowheads indicate the position of nucleus in representative cells. (**b**) Ratio of nuclear YAP intensity to cytoplasmic YAP intensity [+*/*+ , n = 81 cells; *dc/dc* (dilated), n = 43 cells; *dc/dc* (non-dilated), n = 53 cells]. Box plots indicate first quartile, median, and third quartile values. ***p < 0.001, t-test. (**b**) Immunostaining for MLC2, actin, and DAPI in WT and *Camsap3*^*dc/dc*^ kidneys at P22. Arrowheads indicate apical accumulation of MLC2 in cystic tubules.

YAP is a transcriptional activator that regulates cell proliferation^35^ as well as mechanical tension–dependent cell shaping^36^. YAP is activated along with its nuclear localization in kidneys of *Pkd1*-deficient mice, a model of PKD^37^, and Yap activation promotes cell proliferation and tubular dilation in kidneys^28^. We therefore tested whether YAP was also activated in PCTs of *Camsap3*^*dc/dc*^ kidneys. Immunostaining for YAP revealed its cytoplasmic localization in PCTs of WT kidneys. Remarkably, YAP became concentrated in the nuclei of *Camsap3*^*dc/dc*^ PCTs, but only in their dilated portions (Fig. 5a), as confirmed by quantification (Fig. 5b), suggesting that the observed YAP redistribution occurred in response to a change in cell morphology rather than CAMSAP3 dysfunction per se.

YAP is a downstream effector of the Hippo pathway, which is regulated by mechanical cues^38^. In fact, cystic cells in *Camsap3*^*dc/dc*^ kidney appeared stretched, suggesting that they may have been exposed to increased tension. We therefore investigated whether any mechanical changes occurred in concert with cyst formation. Because non-muscle myosin II activation is a tension-sensitive process^39^, we assessed the distribution of myosin light chain 2 (MLC2), which is involved in myosin II activation, in kidneys. Although MLC2 diffused into the cytoplasm in WT tubule cells, it became concentrated near the apical portions of cystic cells in *Camsap3*^*dc/dc*^ kidneys (Fig. 5c arrowhead), suggesting that these cells were subjected to tensile force. Cells in non-dilated PCTs did not show such MCL2 concentration. These findings were in accordance with the idea that YAP enters the nucleus when cells are mechanically stretched^40,41^.

### Changes in PIEZO1 distribution in ***Camsap3***^***dc/dc***^ PCTs

**Figure 6:**
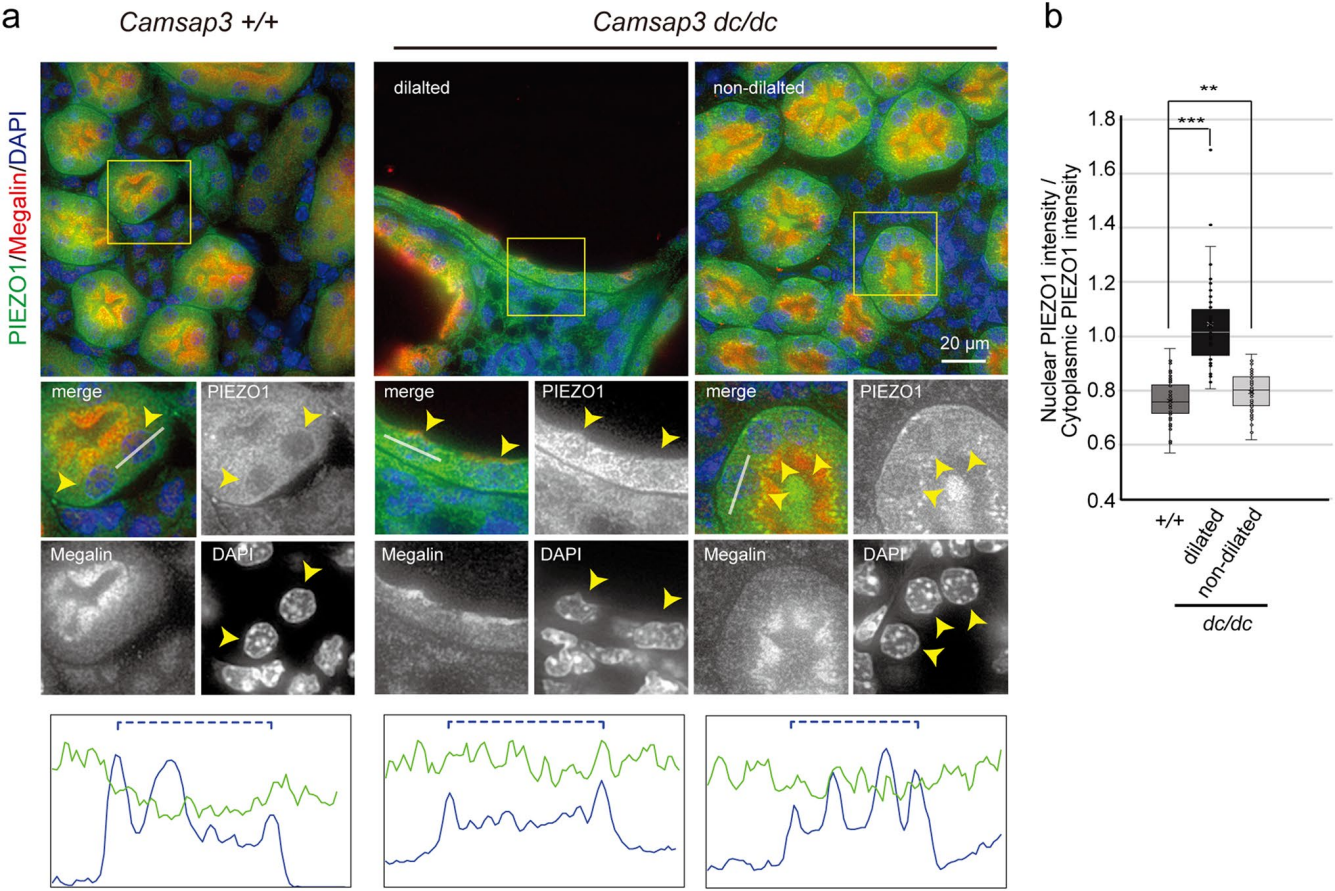
Change in PIEZO1 distribution in *Camsap3*^*dc/dc*^ PCTs. (**a**) Immunostaining for PIEZO1, Megalin, and DAPI in WT and *Camsap3*^*dc/dc*^ kidneys at P22. Fluorescence signals of DAPI and PIEZO1 were scanned along the line drawn in the enlarged images, and the spatial changes in fluorescence intensity are shown in blue for DAPI and green for PIEZO1. Dashed lines indicate the approximate area of the nucleus. Arrowheads indicate the position of the nucleus in representative cells. (**b**) Ratio of nuclear piezo1 intensity to cytoplasmic piezo1 intensity [+*/*+ , n = 66 cells; *dc/dc* (dilated), n = 44 cells; *dc/dc* (non-dilated), n = 81 cells]. Box plots indicate first quartile, median, and third quartile values. ***p < 0.001, **p < 0.01, t-test. See also Figure S4.

To further test whether cystic cells are exposed to stretching force, we examined the subcellular distribution of PIEZO1, a mechanosensitive ion channel^42–45^, as this is a typical marker for mechanical stretch; PIEZO1 localization shifts from the plasma membrane/cytoplasm to the nuclear envelope when it senses stretch signals^46^. Immunostaining for PIEZO1 showed that PIEZO1 mostly localized in the cytoplasm of WT PCT cells, whereas its localization overlapped with the nucleus in cystic cells of *Camsap3*^*dc/dc*^ PCTs (Fig. 6a). Immunostaining for YAP and PIEZO1 in sequential sections also suggested that their nuclear localization coincided at dilated PCTs (Supplementary Fig. S4, arrowheads). On the other hand, in non-dilated *Camsap3*^*dc/dc*^ PCTs, PIEZO1 distribution looked cytoplasmic (Fig. 6a). However, close observations of the immunofluorescence images as well as quantification indicated that its nuclear localization tended to increase to some extent, compared with WT cells (Fig. 6a,b). Thus, unlike YAP, it seems that PIEZO1 begins to be activated prior to the detectable deformation of cells in *Camsap3*^*dc/dc*^ PCTs.

## Discussion

In this study, we found that CAMSAP3 mutation or its loss resulted in dilation of PCT cells, which caused the cross-sections of tubules to take on a cystic appearance. A sign of cystogenesis was already observed as early as E17.5, with a gradual increase of dilation during aging and its eventual extension to the Bowman’s capsule. Epithelial cells in the dilated regions became thinner, exhibiting various cytological defects, as observed in cells of the small intestine of *Camsap3*^*dc/dc*^ mice^10,12^. Some kidney-specific defects were also observed, including the perturbation of mitochondria alignment and basal plasma membrane patterning. These changes coincided with perturbation of the arrangement of microtubules, suggesting that CAMSAP3-mediated microtubule networks are responsible for the normal organization of these subcellular structures in PCT cells, as found in other cell types.

On the other hand, although CAMSAP3 appeared to be expressed in all renal tubule epithelial cells, aberrant structural phenotypes were observed only in PCTs, suggesting the existence of a redundant mechanism(s) to maintain microtubule networks in the absence of CAMSAP3. It is possible that molecules other than CAMSAP3, such as other CAMSAPs, may participate in maintaining the integrity of microtubule networks in these cells. PCT cells had a relatively lower microtubule density than other tubule cells. This probably facilitated cell deformation, as the microtubule networks that would remain after CAMSAP3 loss might have been insufficient to maintain the proper cell architecture. Even in PCT cells, however, mechanisms seem to exist that compensate for the loss of CAMSAP3, as dilation of PCTs did not occur uniformly.

How, then, were PCTs dilated in a regional manner, accompanied by epithelial-cell flattening? Notably, cyst formation became detectable at E17.5, and this timing correlates with the onset of primitive urine production in mice^47^. Therefore, we can hypothesize that ‘urine flow’ is a potential mechanical factor that induces dilation of renal tubules. It is probable that PCT cells that had lost the CAMSAP3-mediated microtubule cytoskeleton were mechanically more susceptible to external forces, that is, more deformable, compared with WT PCT cells. Thus, forces produced by urine flow in the renal tubules might cause tubule dilation, bringing about their flattening. Such forces may not be uniform, resulting in variable degrees of dilation along PCTs. This idea, needless to say, does not exclude other possible mechanisms for PCT dilation.

Our observations demonstrated that PIEZO1 is relocalized from the cytoplasm to the nuclear region in dilated PCT cells of *Camsap3*^*dc/dc*^ kidneys, suggesting that this mechanosensor was activated in response to cell deformation, most likely the ‘stretching’ of cells as reported previously^46^. The stretched state of dilated PCT cells was confirmed by the observation that MLC2 was increased at their cortical regions. Our analysis further indicated that PIEZO1 was already activated to some extent in non-dilated PCT cells, suggesting that even these cells were undergoing mechanical changes despite their normal-looking appearance. Another mechanosensor, YAP, was also activated in PCT cells, but its activation seemed to occur only in stretched cells.

How, then, do these mechanosensitive regulators contribute to cell deformation, as well as cell proliferation which was promoted in the absence of CAMSAP3? It has been shown that the stretch-induced activation of PIEZO1 promotes cell proliferation in epithelial cells^46^, suggesting that this mechanism also works in PCT cells of *Camsap3*^*dc/dc*^ kidneys. This idea is consistent with our observation that enhanced cell proliferation and partial PIEZO1 activation occurred together, even in non-dilated cells of *Camsap3*^*dc/dc*^ PCTs. Concerning YAP, its activation was delayed, not showing a clear correlation with the onset of cell proliferation enhancement. YAP is known not only as a cell growth factor but also as a regulator of mechanical homeostasis, as demonstrated previously^36,48^. It is therefore possible that YAP, which was relocated into the nucleus along with the deformation of PCT cells, might function to regulate their shape, that is, to restore the damaged shape of these cells. Otherwise, YAP might cooperate with PIEZO1 in enhancing the proliferation of dilated cells, as YAP is known to be involved in the formation of polycystic kidneys via enhancement of cell proliferation^28^.

We found that *Camsap3*^*dc/dc*^ mice had significantly lower levels of blood K^+^ and Mg^2+^, suggesting that their kidneys might have suffered from some abnormal functioning. Severe cytological defects that we observed in the apical and basal plasma membranes of PCT cells, where the active transport machinery is located, might be related to this abnormality. However, we cannot exclude the possibility that other portions of renal tubules or other organs could also be functionally defective, inducing this phenotype. In addition, we did not obtain any evidence that the filtering functions of the glomerulus were damaged, as the blood levels of creatinine and urea nitrogen, which are excreted through the glomerulus, were normal.

Disruption of primary cilium biogenesis leads to PKD^24–27^. In the case of *Camsap3* mutation, formation of primary cilia seemed to be unaffected. A recent study showed that loss of CAMSAP3 in nasal multi-ciliated epithelial cells affected the formation of the central pairs of microtubules in the axoneme, causing aberrant motility in motile cilia^49^. Although the primary cilia on epithelial cells are nonmotile and lack the central pairs of microtubules^50^, it remains uncertain whether the primary cilium in the *Camsap3* mutant mice is still functional. Despite this uncertainty, the abnormalities in PKD and *Camsap3* mutants are quite different, as cyst formation occurs uniformly over the entire kidney in the former, whereas it is a local event in the latter. This difference is most likely attributable to the distinct molecular backgrounds of the cyst formation between them.

To summarize, our study reveals that CAMSAP3 is important for maintaining the structure and function of renal tubules at proximal regions, likely through regulation of non-centrosomal microtubule assembly. Further studies will be required, however, for a thorough understanding of how loss of this microtubule organization system interferes with the physiological functions of kidney.

## Materials and methods

All experiments were performed in accordance with relevant guidelines, regulations, and approved by RIKEN Center for Biosystems Dynamics Research’s and/or Waseda University’s Institutional Biosafety Committee.

### Mice

*Camsap3* mutant (*Camsap3*^*dc/dc*^) mice used in this study were generated in our previous study^10^ (accession no. CDB0716K; http://www2.clst.riken.jp/arg/mutant%20mice%20list.html). In those mice, the C-terminal region of *Camsap3*, namely exons 14–17, was removed, and we previously confirmed that mouse cells express a non-functional C-terminally truncated CAMSAP3 (CAMSAP3-dc)^10^. In the present study, N4 and 5 generation mice were used in experiments.

*Camsap3* knockout (*Camsap3*^*–/–*^) mice (accession no. CDB0033E), which lack the entire 22-kb *Camsap3* coding region, were generated with CRISPR/Cas9-mediated genome editing via zygote electroporation^51^. Briefly, two guide RNAs and a single-strand oligodeoxynucleotide (ssODN) were designed to delete the 22-kb region, and an EcoRI site was added for the purpose of recognition for successful insertion in mutant mice. For electroporation, cRNAs, tracrRNA, ssODN and Cas9 protein were introduced into the C57BL/6 zygotes. After electroporation, the zygotes were transferred into pseudopregnant ICR female mice, and the founder mice were screened by PCR and DNA sequencing. Three knockout mice (#4, #10, #23) were obtained, and one of them (#23) had the designed EcoRI knock-in allele. Guide RNA sequences were designed by CRISPRdirect (https://crispr.dbcls.jp). The crRNAs, tracrRNA, and ssODN sequences used for genome editing were synthesized as follows: 5′-cRNA, 5′-UGA UCC CAC ACA UAU GCA GGg uuu uag agc uau gcu guu uug-3′, 3′-cRNA, 5′-CAGCCACCUCUGAUCUGACCguuuuagagcuaugcuguuuug-3′; tracrRNA, 5′-AAACAGCAUAGCAAGUUAAAAUAAGGCUAGUCCGUUAUCAACUUGAAAAAGUGGCACCGAGUCGGUGCU-3′; ssODN, 5′-TAGAACTTACTGTATAGTAATAGCCACAAACTCCATATGCATATGTGAACTCCACCTGAATTCACCTGGCAAGAGATTCCAGACCAGCTCTCCTCTCCTGGGCCTCCTCCACCCCCACAT-3′. The uppercase and lowercase letters indicate coding and auxiliary sequences, respectively. The mouse #23 line was crossed with C57BL/6 three times, and the N3 generation mice were used in experiments.

The experiments using mice were approved by the Institutional Animal Care and Use Committee of the RIKEN Center for Biosystems Dynamics Research or Waseda University, and were performed in accordance with protocols provided by those committees. The study was carried out in compliance with the ARRIVE guidelines: https: //arriveguidelines.org.

### Genotyping

Genotyping for mice was performed by PCR. For *Camsap3*^*dc/dc*^ mice, we used primers described in Toya et al. (2016). For *Camsap3*^*–/–*^ mice, we used the following primers to detect wild-type (WT) (P1/P2, 411 bp) or *Camsap3*^*–/–*^ (P1/P3, 614 bp) allele (Supplementary Fig. S2a,b): P1, 5′- CATGTACCTACCACCATCC-3′; P2, 5′- GGACCCTGGAGAGGCTTAG-3′; P3, 5′- GCAAGGCTGTAGTGAGCC-3′. The P1/P3 region in WT was not amplified under the PCR conditions used.

### Histology: Preparation of frozen tissue sections

Dissected kidney tissue was fixed in 2% paraformaldehyde (Thermo Fisher) / 0.1 M sorbitol (Nacalai Tesque) / PEM buffer for 1 h at room temperature. Recipe for PEM is: 100 mM PIPES (Nacalai Tesque), 5 mM EGTA (Nacalai Tesque), and 5 mM MgCl_2_ (Wako), adjusted to pH 6.9 with 2 M KOH. After fixation, the kidney tissue was rinsed three times with PEM for 10 min. Then, cryoprotection was carried out with sucrose / PEM: 15% sucrose in PEM solution for 2 h at 4 °C, followed by 20% sucrose in PEM solution overnight at 4 °C, and replacement with 30% sucrose in PEM solution for ~ 2 h at 4 °C. The kidney tissue was frozen in OCT compound in an embedding mold (Polysciences, Inc.) in liquid nitrogen and stored at −80 °C. Frozen samples were sliced at 5 µm thickness with a CM1850 Cryostat (Leica). Sections on glass slides were kept at −80 °C until staining.

### Histology: Preparation of paraffin embedded tissue sections

Dissected kidney tissue was fixed in Super Fix solution (Kurabo) for 2 h at room temperature, then transferred to 4 °C overnight. Each sample was exposed to three aqueous ethanol solutions of increasing concentration at room temperature: 30% ethanol for 30 min twice, 50% ethanol for 30 min, and finally 70% ethanol for 30 min. Each sample was processed with an ASP300 Tissue Processor (Leica). Then, the kidney tissue samples were embedded in paraffin wax (Sakura Finetek) with an EG1160 (Leica) and stored at 4 °C. Paraffin-embedded samples were sliced at 6 µm thickness with an RV240 Rotary Microtome (Yamato Kohki). Sections on glass slides were kept at 4 °C until staining.

### Immunofluorescent staining

Frozen kidney sections were immersed in 0.1% Triton X-100 (Nacalai Tesque) / PEM for 10 min at room temperature. Then, samples were permeabilized by applying 0.2% Triton X-100 / PEM for 10 min at room temperature. Once again, samples were immersed in 0.1% Triton X-100 / PEM for 10 min at room temperature. Next, samples were blocked with 3% bovine serum albumin (BSA; Roche) / 0.1% Triton X-100 / PEM for 30 min at room temperature, followed by primary antibody reaction for overnight at 4 °C. Primary antibodies were diluted in 3% BSA / 0.1% Triton X-100 / PEM. For primary antibodies raised in mice, additional blocking with M.O.M. Blocking Reagent (Vector Laboratories) was conducted before blocking in 3% BSA / 0.1% Triton X-100 / PEM. M.O.M. Blocking Reagent was diluted in 0.1% Triton X-100 / PEM and applied to the samples for 30 min at room temperature. After M.O.M treatment, samples were washed with 0.1% Triton X-100 / PEM three times for 5, 10 and 15 min at room temperature. After the primary antibody reaction, samples were washed for 15 min at room temperature with 0.1% Triton X-100 / PEM three times. The secondary antibody reaction was carried out for 2 h at room temperature in the dark. Secondary antibodies were diluted in 3% BSA / 0.1% Triton X-100 / PEM. After the reaction, samples were washed with 0.1% Triton X-100 / PEM three times for 5, 10 and 15 min at room temperature and sealed with VECTASHIELD Mounting Medium (Vector Laboratories).

### Antibodies and reagents

DNA was stained with DAPI solution (Dojindo, D523, 1:1000). F-actin was stained with Alexa Fluor 594 phalloidin (Thermo Fisher, A12381, 1:400). Microtubules were stained with mouse anti-α-tubulin-FITC (DM1A; Sigma Aldrich, F2168, 1:200). The following primary antibodies were used: goat anti-Megalin (P-20; Santa Cruz Biotechnology, sc-16478, 1:200), rabbit anti-NaCl co-transporter (MERCK, AB3533, 1:500), rabbit anti-aquaporin-2 (abcam, AB3274, 1:400), goat anti-SGLT1 (M-19; Santa Cruz Biotechnology, sc-20582, 1:200), rabbit anti-SGLT2 (Novus Biologicals, NBP1-92384, 1:200), mouse anti-ezrin (3C12; abcam, ab4069, 1:500), rabbit anti-giantin (Covance, PRB-114C, 1:700), rabbit anti-aPKC (Santa Cruz Biotechnology, sc-216, 1:400), goat anti-DPP IV (R&D Systems, AF954, 1:100), mouse anti-Na^+^/K^+^-ATPase (abcam, ab7671, 1:100), mouse anti-acetylated tubulin (Sigma Aldrich, T6793, 1:200), rabbit anti-CAMSAP3 (Tanaka et al., 2012, 1:400), mouse anti-tyrosinated tubulin (Sigma Aldrich, T9028, 1:200), rabbit anti-Ki-67 (SP6; abcam, ab16667, 1:250), rabbit anti-YAP (Cell Signaling, #4912, 1:100), rabbit anti-myosin light chain 2 (Cell Signaling, #8505, 1:100), rabbit anti-piezo1 (Novus Biologicals, NBP1-78446, 1:100), rat anti-E-cadherin (^52^, 1:400), and rabbit anti-merlin (Sigma Aldrich, HPA003097, 1:100). The following secondary antibodies were used: donkey Dylight 594–conjugated anti-goat IgG (Jackson, 1:500), goat Alexa Fluor 488/594–conjugated anti-rabbit IgG (Thermo Fisher, A-11034, A-11037, 1:500), goat Alexa Fluor 488/594–conjugated anti-mouse IgG (Thermo Fisher, A-11029, A-11032, 1:500), and goat Alexa Fluor 488–conjugated anti-rat IgG (Thermo Fisher, A-11006, 1:400). The binding specificity of the anti-YAP (Cell Signaling, #4912) and anti-PIEZO1 (Novus Biologicals, NBP1-78446) antibodies used in the study were verified by previous work^46,53^.

### Hematoxylin and eosin (H&E) staining

Glass slides with kidney sections were placed on a 55 °C plate for 30 min to stretch the paraffin. Samples were then immersed in xylene for 15 min twice followed by immersion in a series of 70–100% ethanol: 100% ethanol for 5 min twice, 90% ethanol for 5 min, 80% ethanol for 5 min, and 70% ethanol for 5 min. After rinsing in water for 20 min and in distilled/deionized H_2_O (ddH_2_O) for 1 min, samples were stained in Mayer's hematoxylin solution (Wako) for 5 min. Samples were dipped in ddH_2_O twice, then rinsed with ddH_2_O for 10 min. Samples were again dipped in ddH_2_O and then stained in eosin solution (Merck) for 2 min. For washout, samples were immersed in ddH_2_O for 30 s, followed by immersion in series of 70–100% ethanol and xylene: 70% ethanol for 5 s, 80% ethanol for 5 s, 90% ethanol for 5 s, 100% ethanol for 5 s twice, and xylene for 10 min. Finally, samples were sealed in glycerol.

### Microscopy

Images were acquired with BZ-X710 All-in-one Fluorescence Microscope (Keyence) or LSM880 laser scanning confocal microscope (Zeiss). For BZ-X710, the objective lenses used were Nikon CFI Plan Apo λ 2 × , 10 × , 40 × , 100 × and Nikon CFI Plan Fluor 4 × . Images obtained were processed with BZ-X Analyzer software. The LSM880 was equipped with an Axio Observer Z1 inverted microscope with a Plan-Apochromat 63 × /1.40 NA oil immersion objective lens (Zeiss). Images were processed and analyzed using ImageJ and Adobe Photoshop CC2019.

### Cystic index analysis

Cystic index was analyzed by ImageJ using H&E-stained images of the horizontal section of the kidney. As a measurement of the cyst-containing area, the perimeter of the dilated tubular lumen was traced using the “Freehand selections” function, and its area was measured by the “Measure” function. This work was performed for all cysts within the kidney section, and the areas of all cysts were added together to determine the cyst-containing area. The total kidney area was measured in the same way. The outer circumference of the kidney was traced using the “Freehand selections” function, and its area was assessed. Finally, we calculated the cystic index by dividing the cyst-containing area by the total kidney area.

### Definition of dilated tubules and non-dilated tubules

In this research, tubules with a lumen area of ≧ 500 µm^2^ have been defined as “dilated”, whereas those with a lumen area of < 500 µm^2^ have been defined as “non-dilated". When selecting a “dilated” tubule in *Camsap3*^*dc/dc*^ samples, we first visually picked up a cyst that was not recognized in WT samples and then roughly measured its lumen area using ImageJ. If the value was ≧ 500 µm^2^, it was regarded as “dilated”.

### Electron microscopy

For electron microscopy, P21 male mice were examined. Samples for scanning electron microscopy were prepared according to Takahashi-Iwanaga^54^ with certain modifications, as follows. Mice were anesthetized with isoflurane and perfused with 2% (w/vol) formaldehyde and 2.5% (vol/vol) glutaraldehyde in 0.1 M cacodylate buffer, pH 7.4. Each kidney was excised and cut into cubes that were immersed in the same fixative overnight, and then rinsed with 0.1 M cacodylate buffer. The fixed samples were transferred through a 30% aqueous solution of dimethyl sulfoxide into 60% dimethyl sulfoxide, and then fractured in liquid N_2_. The fractured samples were rinsed with 0.1 M cacodylate buffer and placed in 6 N NaOH for 10–20 min at 60 °C. After maceration in the NaOH solution, each sample was rinsed with 0.1 M cacodylate buffer containing 0.05% Tween 20 and postfixed with 1% OsO_4_ in 0.1 M cacodylate buffer on ice for 2 h. The postfixed samples were: (1) rinsed with ddH_2_O and placed in 1% aqueous thiocarbohydrazide for 20 min at room temperature, and (2) rinsed with ddH_2_O and postfixed again in 1% OsO_4_ in 0.1 M cacodylate buffer for 1 h at room temperature. After repeating steps (1) and (2), the samples were rinsed with ddH_2_O, and stained with 0.5% uranyl acetate solution overnight at room temperature. The tissue blocks were rinsed with ddH_2_O and dehydrated through a graded series of ethanol, transferred to isopentyl acetate, and critical point-dried using liquid CO_2_. The dried specimens were evaporation-coated with osmium and examined with a JSM-5600LV scanning electron microscope at an accelerating voltage of 10 kV. Images were processed with Adobe Photoshop.

For transmission electron microscopy, cubes of dissected kidneys were fixed for at least 2 h in 2% (w/vol) formaldehyde and 2.5% (vol/vol) glutaraldehyde in 0.1 M cacodylate buffer at room temperature. The samples were washed with 0.1 M cacodylate buffer and postfixed with 1% OsO_4_ in 0.1 M cacodylate buffer on ice for 2 h. Samples were then washed with ddH_2_O and stained overnight with 0.5% aqueous uranyl acetate at room temperature. The stained samples were dehydrated with a graded series of ethanol, transferred to propylene oxide, and embedded in Polybed 812 (Polysciences). Ultrathin sections were cut with a diamond knife, double-stained with uranyl acetate and lead citrate, and observed at 100 kV accelerating voltage using a transmission electron microscope (JEM-1010; JEOL) equipped with a charge-coupled device camera (BioScan model 792; Gatan). Images were processed with Adobe Photoshop.

### Urine test and blood test

For comparison with *Camsap3*^*dc/dc*^ mice, WT (+*/*+) and heterozygous (+/*dc*) mice were used as controls in these assays. Heterozygous mice show no signs of Camsap3 dysfunction^10^ and have organized apico-basal arrays of microtubules. Mice were 56–60 weeks old.

Mice were housed in a metabolic cage (TECNIPLAST). Urine samples were collected each day for five consecutive days, and the collected samples were stored at −80 °C. Finally, all five samples for each mouse were mixed so that the final mixture reflected the average condition of each mouse over that period.

For blood collection, mice were administrated 0.02 mL anesthesia per gram body weight. The composition of anesthesia was Domitor (ZENOAQ) 30 µL, Midazolam (Sandoz) 80 µL, Vetorphale (Meiji Seika Pharma) 100 µL, and 0.9% aqueous NaCl to 1 mL. Blood was collected by cardiac puncture. To avoid hemolysis, the syringe was gently inserted and pulled out. Each blood sample was kept at room temperature for 1 h to coagulate. Each sample was then centrifuged (10,000×*g*, 25 °C, 15 min), and serum was collected and stored at −80 °C. All urine and blood samples were analyzed by Oriental Yeast Co.

## Supplementary Information

Supplementary Information
