# Supplementary material for: Cyst formation in proximal renal tubules caused by dysfunction of the microtubule minus-end regulator CAMSAP3
Source: Sci Rep. 2021 Mar 12;11:5857. doi: 10.1038/s41598-021-85416-x (PMC7954811; doi:10.1038/s41598-021-85416-x)
Supplement: Supplementary file 1 — Supplementary Information [file 41598_2021_85416_MOESM1_ESM.pdf]

Cyst formation in proximal renal tubules caused by dysfunction of the microtubule minus-end regulator CAMSAP3

Yuto Mitsuhata[1], Takaya Abe[2], Kazuyo Misaki[3], Yuna Nakajima[1], Keita Kiriya[1], Miwa Kawasaki[4], Hiroshi Kiyonari[2], Masatoshi Takeichi[4,*], Mika Toya[1,4,5,6,*] and Masamitsu Sato[1,6,7]

[1]Laboratory of Cytoskeletal Logistics, Department of Life Science and Medical Bioscience, Graduate School of Advanced Science and Engineering, Waseda University, 2-2 Wakamatsucho, Shinjuku-ku, Tokyo 162-8480, Japan; [2]Laboratory for Animal Resources and Genetic Engineering, RIKEN Center for Biosystems Dynamics Research, Kobe 650-0047, Japan [3]Ultrastructural Research Team, RIKEN Center for Life Science Technologies, Kobe 650-0047, Japan; [4]Laboratory for Cell Adhesion and Tissue Patterning, RIKEN Center for Biosystems Dynamics Research, Kobe 650-0047, Japan; [5]Major in Bioscience, Global Center for Science and Engineering, Faculty of Science and Engineering, Waseda University, 3-4-1 Okubo, Shinjukuku, Tokyo 169-8555, Japan; [6]Institute for Advanced Research of Biosystem Dynamics, Waseda Research Institute for Science and Engineering, Graduate School of Advanced Science and Engineering, Waseda University, 3-4-1 Okubo, Shinjukuku, Tokyo 169-8555, Japan; [7]Institute for Medical-oriented Structural Biology, Waseda University, 2-2 Wakamatsucho, Shinjuku-ku, Tokyo 162-8480, Japan

*To whom correspondence should be addressed: Mika Toya, mikatoya@aoni.waseda.jp; or Masatoshi Takeichi, masatoshi.takeichi@riken.jp

SUPPLEMENTARY FIGURES

Supplementary Figure S1

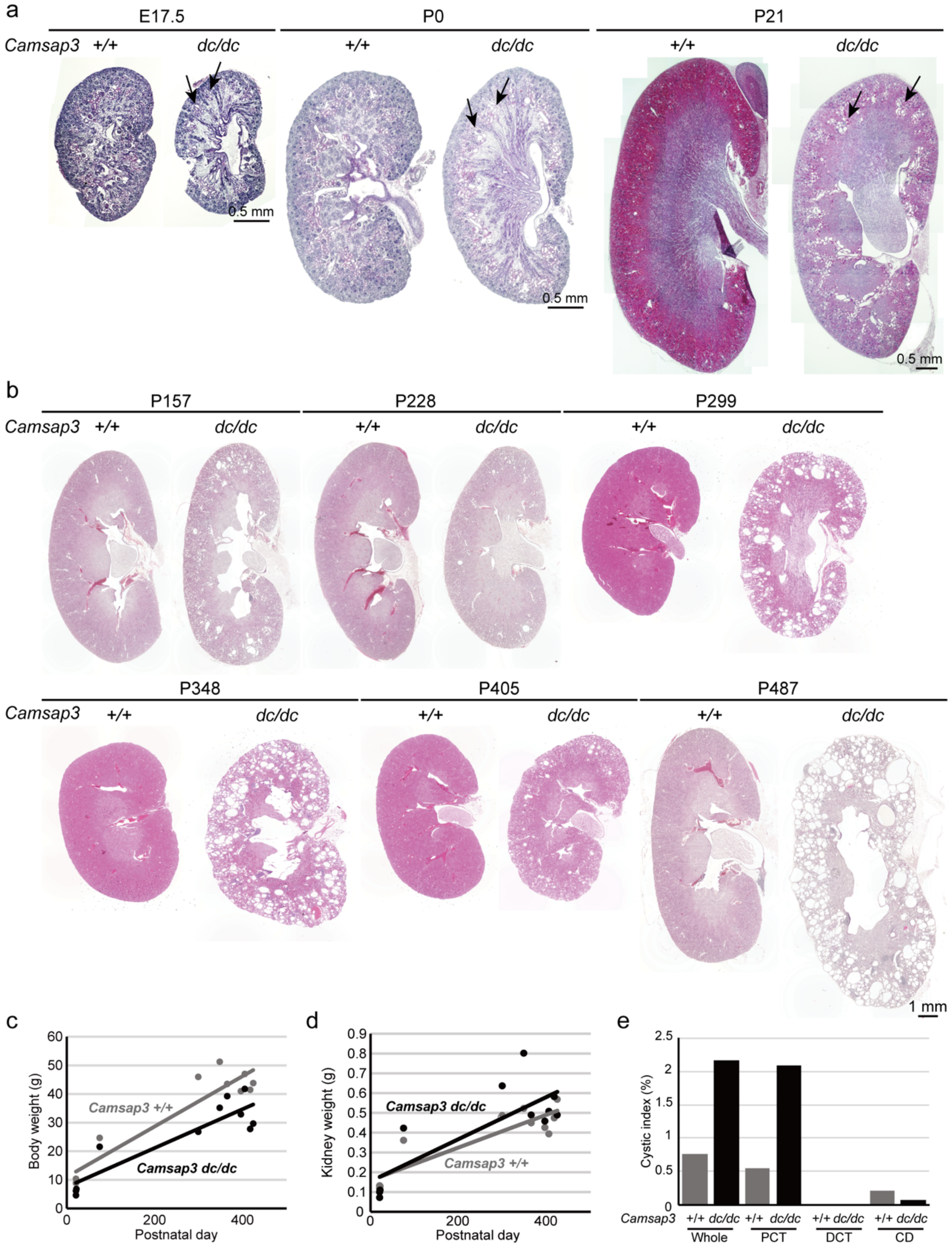

Supplementary Figure S1. Cyst formation in *Camsap3* mutant (*dc/dc*) kidney

(a) H&E staining of WT and *Camsap3*$^{dc/dc}$ kidneys at E17.5, P0, and P21. Cyst formation was observed at E17.5. Arrows denote cystic tubules.

(b) H&E staining of WT and *Camsap3*$^{dc/dc}$ kidneys at P157, P228, P299, P348, P405, and P487.

(c) Body weight of WT and *Camsap3*$^{dc/dc}$ mice shown in Fig. 1D. 11 mice were examined for WT and *Camsap3*$^{dc/dc}$. **(d)** Kidney weight of WT and *Camsap3*$^{dc/dc}$ mice shown in Fig. 1D. **(e)** Cystic index of WT and *Camsap3*$^{dc/dc}$ kidneys obtained using the images shown in Fig. 1E.

Supplementary Figure S2

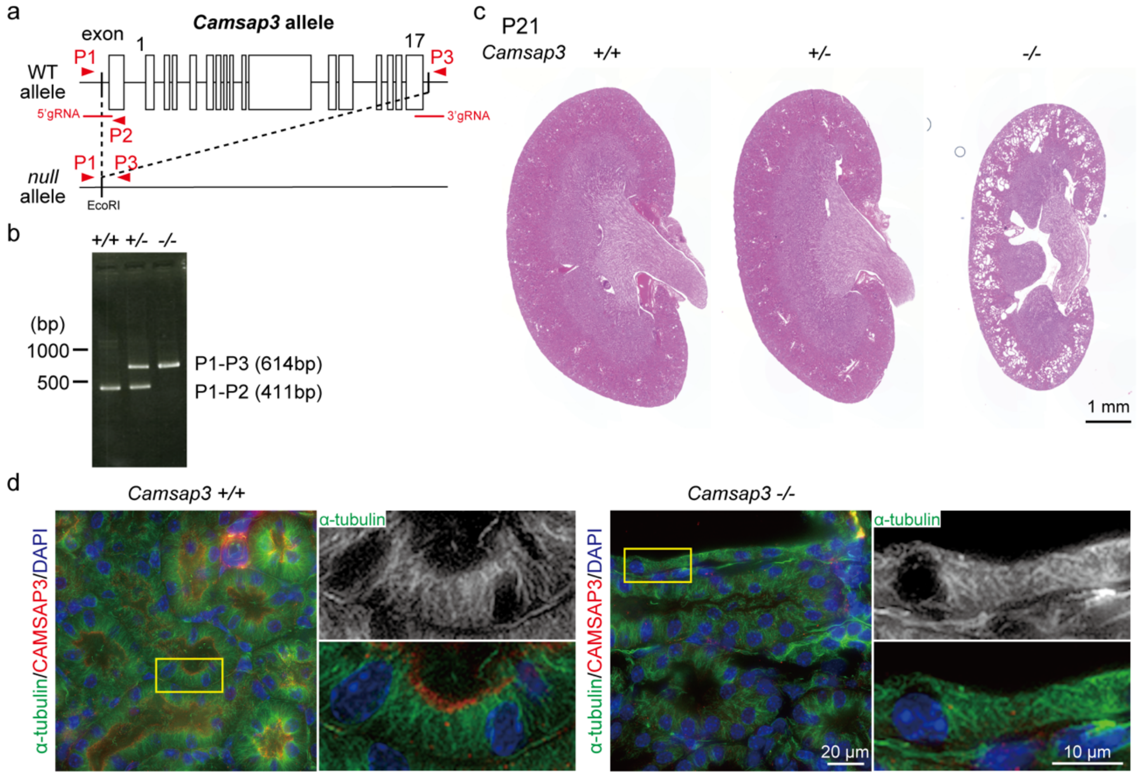

Supplementary Figure S2. Organization of microtubules in WT and *Camsap3 null* (*Camsap3*$^{-/-}$) kidneys

(a) *Camsap3* allele of WT and *Camsap3*$^{-/-}$. Primers 1–3 for genotyping PCR are indicated. **(b)** Genotyping of *Camsap3*$^{-/-}$ mice. For WT, only the region specific for P1–P2 was amplified. **(c)**

H&E staining of *Camsap3* WT, heterozygous (*Camsap3*$^{+/-}$), and *null* (*Camsap3*$^{-/-}$) kidneys at P21. **(d)** Immunostaining for α-tubulin, CAMSAP3, and DAPI in WT and *Camsap3*$^{-/-}$ kidneys at P21. The CAMSAP3 signal was absent from the *Camsap3*$^{-/-}$ (*null*) kidney.

Supplementary Figure S3

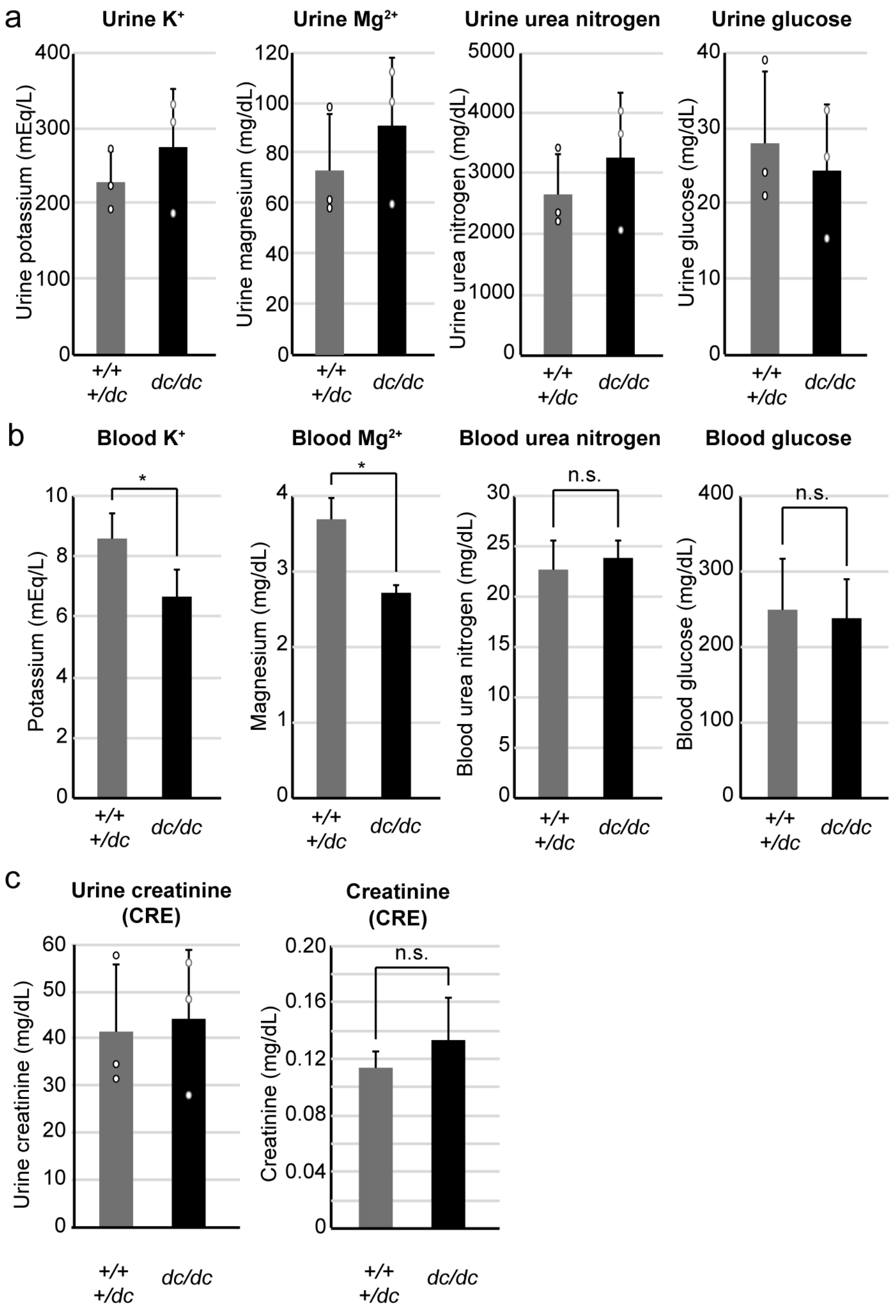

Supplementary Figure S3. *Camsap3*$^{dc/dc}$ mice show physiological defects of the kidney

(a) Levels of K^+, Mg^{2+}, urea nitrogen, and glucose in urine of week 56–60 WT / heterozygous mice (+/+ / +/*dc*) (n = 3 each) and *Camsap3*$^{dc/dc}$ mice (n = 3). Error bars indicate S.D. The excessive outflow of each of K^+, Mg^{2+}, and urea nitrogen was detected in *Camsap3*$^{dc/dc}$ mice. **(b)** Levels of K^+, Mg^{2+}, urea nitrogen, and glucose in blood of week 56–60 WT / heterozygous mice (+/+ / +/*dc*) (n = 3 each) and *Camsap3*$^{dc/dc}$ mice (n = 3). Error bars indicate S.D. *$p < 0.05$, t-test. The *Camsap3*$^{dc/dc}$ mice had significantly lower levels of K^+ and Mg^{2+}. **(c)** Levels of urine creatinine (left) and blood creatinine (right) of week 56–60 WT / heterozygous mice (+/+ / +/*dc*) (n = 3 each) and *Camsap3*$^{dc/dc}$ mice (n = 3). Error bars indicate S.D.

Supplementary Figure S4

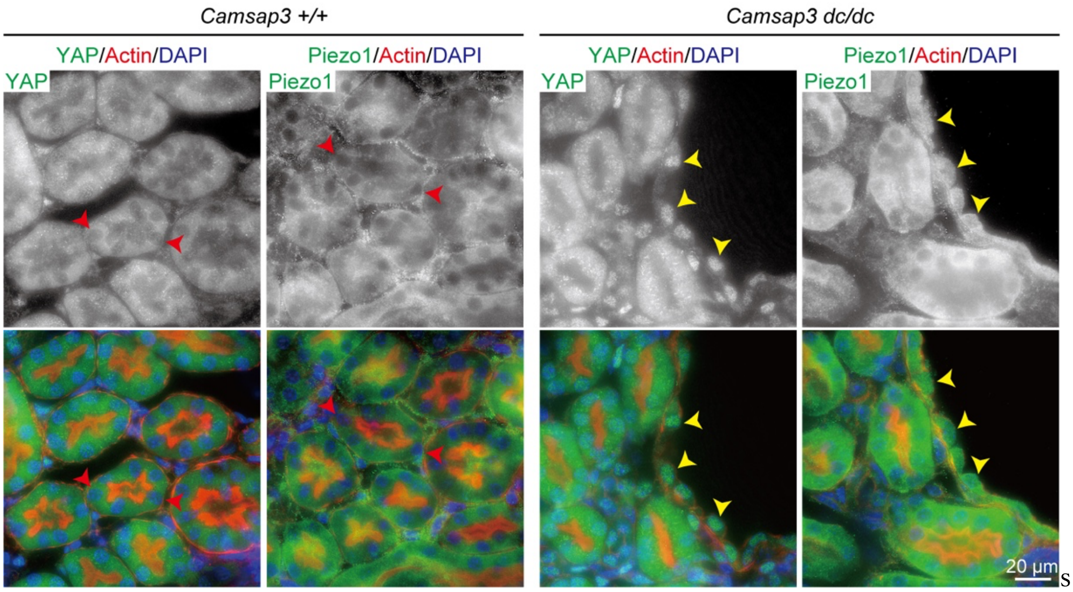

Supplementary Figure S4. Nuclear localization of YAP and Piezo1 coincides at dilated PCTs

Immunostaining for YAP and PIEZO1 along with actin and DAPI in sequential sections in WT and *Camsap3*$^{dc/dc}$ kidneys at P22. Red arrowheads indicate WT cells lacking nuclear accumulation of YAP. Yellow arrowheads indicate *Camsap3*$^{dc/dc}$ cystic cells with nuclear accumulation of YAP.
